# Dehydroepiandrosterone Antagonizes Pain Stress-Induced Suppression of Testosterone Production in Male Rats

**DOI:** 10.3389/fphar.2018.00322

**Published:** 2018-04-16

**Authors:** Qiqi Zhu, Fei Ge, Xiaoheng Li, Hou-Sheng Deng, Miao Xu, Tiao Bu, Jingyang Li, Yiyan Wang, Yuanyuan Shan, Ren-Shan Ge, Ming Yao

**Affiliations:** ^1^Department of Anesthesiology of the Second Affiliated Hospital and Yuying Children’s Hospital, Wenzhou Medical University, Wenzhou, China; ^2^General Hospital of Guangzhou Military Command of PLA, Guangzhou, China; ^3^Department of Neonatology, Xi’an No.4 Hospital, Xi’an, China

**Keywords:** acute stress, pain, Leydig cell, steroidogenic enzymes, DHEA, corticosterone, 11β-hydroxysteroid dehydrogenase 1

## Abstract

**Background:** Leydig cells secrete the steroid hormone, testosterone, which is essential for male fertility and reproductive health. Stress increases the secretion of glucocorticoid [corticosterone, (CORT) in rats] that decreases circulating testosterone levels in part through a direct action on its receptors in Leydig cells. Intratesticular CORT level is dependent on oxidative inactivation of CORT by 11β-hydroxysteroid dehydrogenase 1 (HSD11B1) in rat Leydig cells. Pain may cause the stress, thus affecting testosterone production in Leydig cells.

**Methods:** Adult male Sprague–Dawley rats orally received vehicle control or 5 or 10 mg/kg dehydroepiandrosterone (DHEA) 0.5 h before being subjected to pain stimulation for 1, 3, and 6 h. In the present study, we investigated the time-course changes of steroidogenic gene expression levels after acute pain-induced stress in rats and the possible mechanism of DHEA that prevented it. Plasma CORT, luteinizing hormone (LH), and testosterone (T) levels were measured, and Leydig cell gene expression levels were determined. The direct regulation of HSD11B1 catalytic direction by DHEA was detected in purified rat Leydig, liver, and rat *Hsd11b1*-transfected COS1 cells.

**Results:** Plasma CORT levels were significantly increased at hour 1, 3, and 6 during the pain stimulation, while plasma T levels were significantly decreased starting at hour 3 and 6. Pain-induced stress also decreased *Star, Hsd3b1*, and *Cyp17a1* expression levels at hour 3. When 5 and 10 mg/kg DHEA were orally administered to rats 0.5 h before starting pain stimulation, DHEA prevented pain-mediated decrease in plasma T levels and the expression of *Star, Hsd3b1*, and *Cyp17a1* without affecting plasma CORT levels. DHEA was found to modulate HSD11B1 activities by increasing its oxidative activity and decreasing its reductive activity, thus decreasing the intracellular CORT levels in Leydig cells.

**Conclusion:** Stress induced by acute pain can inhibit Leydig cell T production by upregulation of corticosterone. DHEA can prevent the negative effects of excessive corticosterone by modulating HSD11B1 activity.

## Introduction

Many stressors, such as noise, pain, diseases, and pollution, are present in our daily life (Munck et al., 1984; Chrousos and Gold, 1992; de Quervain et al., 2017). Pain is a potential stressor, although it is not completely clear yet how stress and pain are related.

Stress induced the elevation of circulating glucocorticoid (GC) level (Munck et al., 1984; Chrousos and Gold, 1992). Increased GC levels disrupted the male reproductive axis (Baldwin, 1979; Suter and Schwartz, 1985). It has been demonstrated that excessive GC levels significantly reduced testosterone (T) level (Phillips et al., 1989). GCs directly inhibited T production by Leydig cells, which are responsible for 95% of plasma T levels in males (Hales and Payne, 1989; Monder et al., 1994b; Orr et al., 1994). Further studies showed that GCs directly inhibited the transcription of genes encoding the rate-limiting cholesterol transporter, the steroidogenic acute regulatory protein (STAR, gene *Star*), and T biosynthetic enzymes, including cholesterol side-chain cleavage enzyme (CYP11A1, gene *Cyp11a1*), 3β-hydroxysteroid dehydrogenase 1 (HSD3B1, gene *Hsd3b1*), 17α-hydroxylase/20-lyase (CYP17A1, gene *Cyp17a1*), and 17β-hydroxysteroid dehydrogenase 3 (HSD17B3, gene *Hsd17b3*) (Bambino and Hsueh, 1981; Hales and Payne, 1989; Payne and Sha, 1991; Agular et al., 1992; Orr et al., 1994; Agular and Vind, 1995). It is important to note that the harmful effects of stress on Leydig cells do not necessarily result from the GC receptor (NR3C1, gene *Nr3c1*) binding exclusively, since other factors such as testicular opioids (Akibami and Mann, 1996) or nitric oxide (NO) generated by NO synthase (NOS), including testicular NOS2 (gene *Nos2*) (Kostic et al., 1998), might also be involved.

Leydig cells produce T, which stimulates differentiation of the male phenotype and spermatogenesis in the testes. In rat Leydig cells, the T biosynthetic pathway started from the precursor, cholesterol. Cholesterol in lipoprotein-bound form was transported via a high-density lipoprotein receptor, also called scavenger receptor class B member 1 (SCARB1, encoded by *Scarb1*), into Leydig cells, where cholesterol was released for storage as cholesterol esters or use in steroidogenesis. In Leydig cells, after stimulation by pituitary-secreted luteinizing hormone (LH), cholesterol was mobilized to the inner mitochondrial membrane via STAR (Stocco, 2001), where it was converted into pregnenolone by CYP11A1 (Stocco, 2001). Pregnenolone can diffuse out of the mitochondrion into the membrane of the smooth endoplasmic reticulum, which contains HSD3B1 and other steroidogenic enzymes (CYP17A1 and HSD17B3). Upon conversion of pregnenolone into progesterone by dehydrogenation, two additional enzymes, CYP17A1 and HSD17B3 converted progesterone sequentially to 17-hydroxyprogesterone, androstenedione, and ultimately to T (Ge and Hardy, 2007).

The T biosynthesis by Leydig cells is predominantly regulated by LH, which contains two subunits with LH β chain (LHB, encoded by *Lhb*), the rate-limiting subunit. LH binds to the LH receptor (LHCGR, encoded by *Lhcgr*) on the surface of the Leydig cell to activate adenylyl cyclase, leading to a cascade to stimulate T production. In the pituitary, LH secretion was positively regulated by gonadotropin-releasing hormone (GnRH), which bound to its receptor (GNRHR, encoded by *Gnrhr*), and negatively regulated by estradiol, which bound to its receptor α (ESR1, encoded by *Esr1*) (Akingbemi et al., 2004).

Leydig cells possessed NR3C1 (Schultz et al., 1993; Ge et al., 1997b). In this case, GCs can act on Leydig cells to elicit their adverse effects. Leydig cells had a protective mechanism against the over-abundance of active GCs (the corticosterone, or CORT, in rats) via 11β-hydroxysteroid dehydrogenase isoform 1 (HSD11B1, encoded by *Hsd11b1*). This enzyme acted as a primary oxidase and inactivated biologically active CORT into inert 11-dehydrocorticosterone (DHC), thus controlling the level of active CORT in Leydig cells (Ge et al., 1997a,b, 2005a,b).

In our preliminary study, we demonstrated that pain can significantly elevate CORT levels and block T biosynthesis in Leydig cells. There were still few therapeutic drugs for stress-induced T reduction, although in the animal model, the blockade of NR3C1 using its antagonist RU486 can partially restore the T levels. In the present study, we reported that dehydroepiandrosterone (DHEA) can prevent pain-induced hypogonadism via targeting HSD11B1.

## Materials and Methods

### Chemicals

Rat LH standard NIDDK-r-LH-I9 was obtained through the National Hormone and Pituitary Program (United States). ^3^H-CORT, ^3^H-hydroxycholesterol, ^3^H-pregnenolone, ^3^H-progesterone, ^3^H-androstenedione, and ^3^H-T were purchased from DuPont-New England Nuclear (Boston, MA, United States). ^3^H-DHC was prepared as previously described (Lakshmi and Monder, 1985). The CORT antiserum B3-163 was obtained from Endocrine Sciences (Calabasas, CA, United States). NAD^+^ and NADPH were purchased from Sigma (St. Louis, MO, United States). RU486 was from Roussel (UCLAF, France). DHEA, 2-hydroxypropyl-cyclodextrin, etiocholan-3β-ol-17-one, M-199, DMEM, and Ham’s F-12 medium were purchased from Sigma (St. Louis, MO, United States).

### Animals

Adult male Sprague–Dawley rats (90-day-old) were purchased from Shanghai Animal Center (Shanghai, China), and housed under controlled environmental conditions (temperature 22 ± 2°C; 12:12 h light:dark, with lights on from 0600 to 1800 h). All animals were handled to become adapted for at least 3 weeks prior to the beginning of the experiment. All animal procedures were performed in accordance with the National Institutes of Health Guide for the Care and Use of Laboratory Animals according to protocols approved by the Animal Care and Use Committee of Wenzhou Medical University.

### Acute Pain Stimulation

Experiment 1 (acute pain stimulation): Thirty-six 90-day-old male Sprague–Dawley rats were randomly divided into two groups: the unstressed control and the stress. Rats in the stress group orally received 0.5 ml vehicle control (a solvent for DHEA) and 0.5 h later they were subjected to pain simulation. DHEA was first dissolved in ethanol and subsequently diluted with the vehicle, 45% aqueous 2-hydroxypropyl-cyclodextrin, to attain the needed concentrations. The thermal pain stimulation was performed using a radiant heat. Rats were placed in clear plastic cages on an elevated glass plate. A radiant thermal stimulator was focused onto the plantar surface of the hind paw through the glass plate. The heat stimulation was performed for 2 min every 5 min for six times at hour 1, 3, and 6. Then, rats were placed back in the original cage. The pain stimulation began at 9 AM and the treatment durations were 1, 3, and 6 h (*n* = 6 per time point). Control animals orally received vehicle control and then were left undisturbed in their cages for the duration of the experiment and sampled at the same time point. At the end of each stress period, animal were euthanized by CO_2_, trunk blood was collected in tubes containing heparin and centrifuged at 500 × *g*, and the plasma were stored at -20°C until assay. Testes and pituitaries were removed and stored at -80°C. Testes were used for analysis of Leydig-cell-specific gene and protein expression levels. The pituitaries were used for analysis of pituitary-specific gene expression.

Experiment 2 (DHEA treatment): Eighteen rats were randomly divided into three groups: the stress control (0 mg/kg DEHA), 5 mg/kg DHEA, and 10 mg/kg DHEA. Rats were gavaged 0, 5, or 10 mg/kg DEHA and then 0.5 h later they were subjected to a 3-h period of pain stimulation as above.

### Intratesticular Treatment of RU486

Experiment 3 (NR3C1 antagonist study): To investigate whether NR3C1 acted in pain-induced T decline during the course of pain, the specific blocker of NR3C1, RU486, was administered *in vivo* via intratesticular injection 0.5 h prior to the pain stimulation session. We selected the RU486 dose as 16 μg based on our previous observation (Lin et al., 2014). RU486 was first dissolved in ethanol and subsequently diluted with the vehicle, 45% aqueous 2-hydroxypropyl-cyclodextrin to attain the needed concentrations and the final concentration of ethanol was 0.8%, which did not affect Leydig cell function (Dong et al., 2004). Twenty-four animals (*n* = 6 per group) were divided into four groups: (1) control no-pain group (injected with vehicle); (2) no-pain group with injection of RU486; (3) pain group (injected with vehicle); and (4) pain group with injection of RU486. Rats of pain groups were subjected to pain stimulation as above during the course of a 6-h period. At the end of the session, animals were euthanized by CO_2_, testes were taken, and interstitial fluids were prepared according to the previously described method (Maddocks and Sharpe, 1989).

### Interstitial Fluid Collection

Testes were taken after treatment and interstitial fluids were prepared according to the previously described method (Lin et al., 2014). Briefly, five holes were punctured in the rat testis using a G27 needle and the testis was placed into a centrifuge tubule, and the testis was centrifuged at 800 × *g* for 10 min to collect the interstitial fluid.

### Leydig Cell Isolation

Experiment 4 (Leydig cell stimulation study): To investigate whether Leydig cell steroidogenesis was affected after pain stimulation, animals (*n* = 6 per group) were subjected to no pain or pain stimulation for 3 h as above, since at the end of this time point, plasma T levels were significantly decreased. At the end of the pain stimulation session, animals were euthanized by CO_2_, and trunk blood was collected for hormonal assays. Leydig cells were harvested for direct measurement of T production and steroidogenic enzyme activities *ex vivo* as follows:

Leydig cells were purified as previously described (Salva et al., 2001). In brief, testes were removed, decapsulated, and dispersed in 10 ml of medium 199 with 0.25 mg/ml collagenase in a shaking water bath at 34°C for 10 min. To terminate collagenase dispersion, 1% bovine serum albumin (BSA), M-199 buffered with 15 mM HEPES and 4 mM sodium bicarbonate, and soybean trypsin inhibitor was added to dilute the original suspension. Seminiferous tubules were allowed to settle, and the supernatant was collected by aspiration. The centrifuge tubes containing the settled seminiferous tubules were refilled with 1% BSA. The cells were pelleted by centrifugation at 800 × *g* for 20 min at 4°C and then fractionated using a continuous Percoll gradient (55% Percoll in Hanks balanced salt solution). Leydig cells were recovered starting at a density of 1.07 mg/ml. Leydig cells were washed using M-199 and were pelleted at 800 × *g* for 10 min at 4°C. The Leydig cell purity was >95% (*n* = 6), as determined by histochemical staining for HSD3B1 using 0.4 mM etiocholan-3β-ol-17-one as the enzyme substrate and NAD+ as cofactor according to a previously described method (Payne et al., 1980).

### Steroidogenesis *ex Vivo*

The steroidogenesis of isolated adult Leydig cells was conducted *ex vivo* as previously described (Lin et al., 2014). Leydig cells were incubated at a concentration of 0.1 × 10^6^ cells/ml in the culture medium consisting of DMEM and F-12 medium buffered with 15 mM HEPES and 14 mM NaHCO_3_, and containing 1% BSA for 3 h at 34°C in a shaking water bath (75 rpm). Incubations of quadruple samples were conducted in medium alone (Basal) or in medium plus a maximally stimulating concentration of ovine LH (100 ng/ml), 20 μM 22-R-hydroxysteroid cholesterol (22OHC). LH was used to examine the Leydig cell functionality after it bound to LHCGR. 22OHC was used to examine the steroidogenic enzyme activity of CYP11A1 or beyond. At the end of 3 h, the samples were centrifuged at 500 × *g*. Supernatants were used to measure T levels by RIA.

### Liver Cell Isolation

Liver parenchymal cells were isolated according to the previously described method (Ge et al., 1997a). Livers of male adult Sprague–Dawley rats were perfused *in situ* with a calcium-free buffer, then dispersed by a solution containing 0.05% collagenase, and parenchymal cells were purified by density gradient centrifugation in Percoll. The purity of parenchymal cells in the final suspension was assessed by judging the uniformity of cell size in hematocytometer counts and was typically over 95%.

### RIA of CORT, LH, and T

Plasma CORT level was measured according to a previously described method (Spencer et al., 1996). Briefly, plasma samples (20 μl) were diluted in 1 ml of 0.01 M PBS and were heated at 60 °C for 1 h in order to inactivate corticosteroid-binding globulin. The heat inactivated samples (100 μl in triplicate) were incubated overnight with a mixture of rabbit CORT antiserum and ^3^H-CORT. CORT standards (10–2000 pg/100 μl) were assayed in parallel. Bound steroid was separated from free steroid by mixing with dextran-coated activated charcoal followed by centrifugation. The bound supernatant was put into a bottle with a scintillation cocktail and the relative amount of radioactivity was determined by a liquid scintillation counter (Packard, Meriden, CT, United States). Assay sensitivity was 10 pg of CORT per assay tube. The interassay coefficient of variability was calculated and it was 7% (*n* = 4).

Plasma LH level was measured by RIA according to a previously described method (Kumar et al., 1992). Briefly, plasma samples were incubated with ^125^I rat LH and immunoglobulin-G (IgG) antiserum. LH reference standards (NIDDK-rLF-RP-3) were used. The lower limit of detection for this assay is 0.12 ng/ml and LH values are expressed in relation to the RP-3 standards. The intraassay and interassay coefficients of variation were 5 and 10%, respectively (Kumar et al., 1992).

Plasma, testicular fluid, and medium T levels were measured with a tritium-based ^3^H-T RIA according to a previously described method (Cochran et al., 1981). Briefly, plasma samples (50 μl) or intratesticular fluid samples (5 μl) or medium samples (1–50 μl) were incubated overnight with a mixture of rabbit T antiserum and ^3^H-T at 4°C. T standards (10–2000 pg/100 μl) were assayed in parallel. Bound steroid was separated from free steroid by mixing with dextran-coated activated charcoal followed by centrifugation. The bound supernatant was put into a bottle with scintillation cocktail and the relative amount of radioactivity determined as above. The interassay coefficient of variability was calculated and it was 7.5% (*n* = 4).

### Real-Time PCR (qPCR)

Total RNAs were extracted from the pituitaries and testes using a Trizol kit (Invitrogen, Carlsbad, CA, United States) according to the manufacturer’s instruction. Twelve genes in the testes and four genes in the pituitary were analyzed and their primers were listed in Supplementary Table S1. The relative mRNA levels of target genes were normalized to *Rps16* (the house-keeping gene as an internal control). The RNA was reversely transcribed using a random hexamers and MMLV reverse transcriptase (Promega, San Luis Obispo, CA, United States) according to the manufacturer’s instruction. Real-time PCR (qPCR) was carried out in a 25-μl volume with SYBR Green kit (Promega, San Luis Obispo, CA, United States) according to the manufacturer’s instruction. Reactions were carried out and fluorescence was detected on a Bio-Rad qPCR system (Bio-Rad Laboratories, Inc., Hercules, CA, United States). The Ct values were recorded and the standard curve was generated and the gene expression levels were calculated according to the standard curve as previously described (Lin et al., 2014).

### Western Blotting Analysis

STAR and LHCGR levels in the testes were measured using Western blotting analysis as previously described (Ge et al., 1997a). In brief, the homogenized testis samples (10 μg protein) were boiled in equal volumes of sample loading buffer, a Tris-HCl buffer (pH 6.8) containing 20% glycerol, 5% SDS, 3.1% dithiothreitol, and 0.001% bromophenol blue. The samples were then electrophoresed on 10% polyacrylamide gels containing SDS. Proteins were then electrophoretically transferred onto the nitrocellulose membranes, and after a 30-min exposure to 10% non-fat milk to block non-specific binding, the membranes were incubated with a 1:1000 dilution of a rabbit polyclonal anti-STAR antibody (Pterosaur Biotech, Hangzhou, Zhejiang, China) or a goat polyclonal anti-LHCGR antibody from Santa Cruz (Santa Cruz, CA, United States). The membranes were then washed and incubated with a 1:2500 dilution of anti-rabbit antiserum or anti-goat IgG secondary antibody (1:5000, Bioword, United States) that was conjugated to the horseradish peroxidase. The washing step was repeated, and immunoreactive bands were visualized with the ECL chemiluminescence kit from Amersham (Arlington Heights, IL, United States). The β-Actin (ACTB, Cell Signaling Technology, Danvers, MA, United States; dilution 1:1000) was used as control. Protein levels were measured by densitometry of the films.

### Construction of Rat HSD11B1 Plasmid and Transfection

Rat HSD11B1 expression plasmids were constructed to express rat HSD11B1 using *Hsd11b1* cDNA in pcDNA I expression vector from its original rat *Hsd11b1* vector (p11DH-I) (Agarwal et al., 1989). The *Escherichia coli* transformants carrying an insert of either rat *Hsd11b1* were selected by colony hybridization, and a clone with the inset in the correct orientation relative to the vector T7Q promoter was identified by restriction enzyme mapping. All transfections were carried out on 80% contiuent cultures in 12-well plates. Aliquots of 1 μg rat *Hsd11b1* pcDNA I were transfected into mammalian COS1 cells to make COS1-*Hsd11b1* cells with the FuGENE Transfection Reagent (Roche) according to the manufacturer’s protocol. Cells were allowed to grow for 24 h in media containing 10% fetal bovine serum. Then, the media were removed, and cells were harvested for HSD11B1 assay.

### Analysis of Steroidogenic Enzyme Activities

CYP11A1, HSD3B1, CYP17A1, and HSD17B3 activities in intact Leydig cells *ex vivo* were measured according to the previously described method (Ge and Hardy, 1998). In brief, the radiolabeled substrates (1 mCi) for CYP11A1 (^3^H-hydroxychloesterol, 81.9 Ci/mmol, 2 μM), HSD3B1 (^3^H-pregnenolone, 12.6 Ci/mmol, 2 μM), CYP17A1 (^3^H-progesterone, 86.0 Ci/mmol, 2 μM), and HSD17B3 (^3^H-androstenedione, 90.0 Ci/mmol, 2 mM) were added. Reactions were initiated by adding to the reaction medium an aliquot of 0.1 × 10^6^ Leydig cells. The reaction mixtures, conducted in triplicate, were maintained at 34°C in a shaking water bath (75 rpm) for 10 min. The radioactive products were produced. Reactions were terminated by adding ice-cold ether, and steroids were rapidly extracted. The ether layer was dried up under a nitrogen gas. The radioactivity was measured by System 200/AC3000 radioactive scanned (Bioscan, Washington, DC, United States). The activity of HSD3B1 was determined by measuring the conversion of pregnenolone into progesterone. The activity of CYP17A1 was determined by measuring conversion of progesterone into 17α-hydroxyprogesterone and androstenedione. The activity of HSD17B3 was determined by measuring the conversion of androstenedione into T. The steroids were separated on thin layer chromatographic plates in chloroform-methanol (97:3, v/v) for HSD3B1 and HSD17B3 assays, chloroform-ether (7:1, v/v) for CYP17A1 assay. The activity of CYP11A1 was determined by measuring the conversion of side-chain labeled ^3^H-hydroxycholesterol to radioactive 4-hydroxyl-4-methyl-pentanoic acid. The mixture was extracted twice with 2 ml chloroform and mixed with neutral alumna to remove non-metabolized substrate, and an aliquot was removed for measurement by liquid scintillation counting at a scintillation counter (Packard, Meriden, CT, United States).

### Preparation of Rat Testis Microsomal Fraction for Measurement of HSD11B1 Activity

Leydig cell microsomal fraction was prepared from rat testis as described previously (Chen et al., 2009), because Leydig cells expressed the highest HSD11B1 activity (Monder et al., 1994a). In brief, Leydig cells isolated from 90-day-old male Sprague–Dawley rats (control) were homogenized in ice-cold 0.01 mM PBS containing 0.25 mM sucrose and centrifuged at 700 × *g* for 30 min. The supernatants were transferred and centrifuged at 10,000 × *g* for an additional 30 min to get rid of mitochondria. The remaining supernatants were further centrifuged at 105,000 × *g* for 1 h twice. Microsomal pellets were resuspended by adding 1 ml ice-cold PBS and homogenized by a glass homogenizer. Protein concentrations in the microsomal fractions were measured by Bio-Rad Protein Assay Kit (cat #500-0006; Bio-Rad, Hercules, CA, United States) according to manufacturer’s protocol. Microsomal fractions were used for the measurement of HSD11B1.

### HSD11B1 Assay in Intact Cells and the Microsomal Fractions

HSD11B1 activity was assayed according to the previously described method (Ge and Hardy, 2000). HSD11B1 oxidative activity was measured by adding 25 nM ^3^H-CORT into 0.1 × 10^6^ Leydig, liver, and COS1-*Hsd11b1* cells using the endogenous NADP^+^. HSD11B1 reductive activity was measured by adding 25 nM ^3^H-DHC into 0.1 × 10^6^ Leydig, liver, and COS1-*Hsd11b1* cells using the endogenous NADPH. For HSD11B1 reductive activity in the microsomes, the enzyme assay tube contained 25 nM DHC spiked with 60,000 dpm of ^3^H-DHC, 0.2 mM NADPH, and 10 μg rat microsomal fractions in 0.25 ml PBS. The reaction was initiated by adding microsomal fractions. For testing the potential of DHEA to affect HSD11B1 activity, DHEA was dissolved in ethanol, with a final ethanol concentration of 0.4%, and this concentration did not affect rat HSD11B1 activity. The reaction was started by incubating the reaction mixture at 34°C in the 75 rpm shaking water bath. The reactions were stopped by adding 2 ml ice-cold ether. The reaction mixtures were mixed with ether thoroughly by vortexing, and the steroids were extracted. The ether layer was transferred into a new glass tube and was dried up under nitrogen gas. The steroids were plotted onto a thin layer chromatographic plate and separated chromatographically in chloroform and methanol (90:10, v/v). The plate was dried up, and the radioactivity on the plate was measured using Bioscan scanning radiometer (Bioscan, Inc., Washington, DC, United States) according to the manufacturer’s instructions. The percentage conversion of 11DHC to CORT or cortisone to cortisol was calculated by dividing the radioactive counts identified by the total counts.

### Determination of Half Maximum Effective Concentrations (EC_50_) and Mode of Action

The EC_50_ value was determined by adding either 25 nM CORT or DHC with 0.2 mM cofactor and various concentrations of DHEA in 250 μl reaction buffer (0.1 mM PBS) containing rat enzyme in Leydig cell microsomes as previously described (Guo et al., 2012). For the activation of HSD11B1 oxidase by DHEA, EC_50_ was calculated. For the inhibition of HSD11B1 reductase, IC_50_ was calculated. The method for the mode of action was similar to the measurement of EC_50_ except for adding various concentrations of 11DHC or CORT as previously described (Wu et al., 2017).

### Molecular Docking Simulation Analysis

The crystal structure of recombinant human HSD11B1, which has a complex with NADP^+^ and HSD11B1 inhibitor adamantane sulfone (PDB id 2ILT; Sorensen et al., 2007) was used as a molecular docking target for ligand cortisone as well as DHEA. However, the crystal structure of rat HSD11B1 was not available. The structures of cortisone and DHEA were obtained from PubChem^1^ as ligands. Molecular docking simulation calculations were performed with SwissDock, a docking algorithm based on the docking software EADock DSS (Grosdidier et al., 2011). The free energy was calculated. The docked file was visualized using a program Chimera 1.1.1 (San Francisco, CA, United States). The best model for ligand fit was selected.

### Statistical Analysis

The data were analyzed by Student’s *t*-test between control and pain stimulation animals to determine a significant difference between the two groups at each time point during the course of pain. One-way ANOVA followed by *ad hoc* Tukey’s multiple comparison to identify the significant differences between two groups when three and more groups were compared. All data are expressed as mean ± SEM. Differences were regarded as significant at ^∗^*P* < 0.05, ^∗∗^*P* < 0.01, ^∗∗∗^*P* < 0.001.

## Results

### Pain Increases Plasma CORT Levels but Decreases Plasma T Levels

**FIGURE 1:**
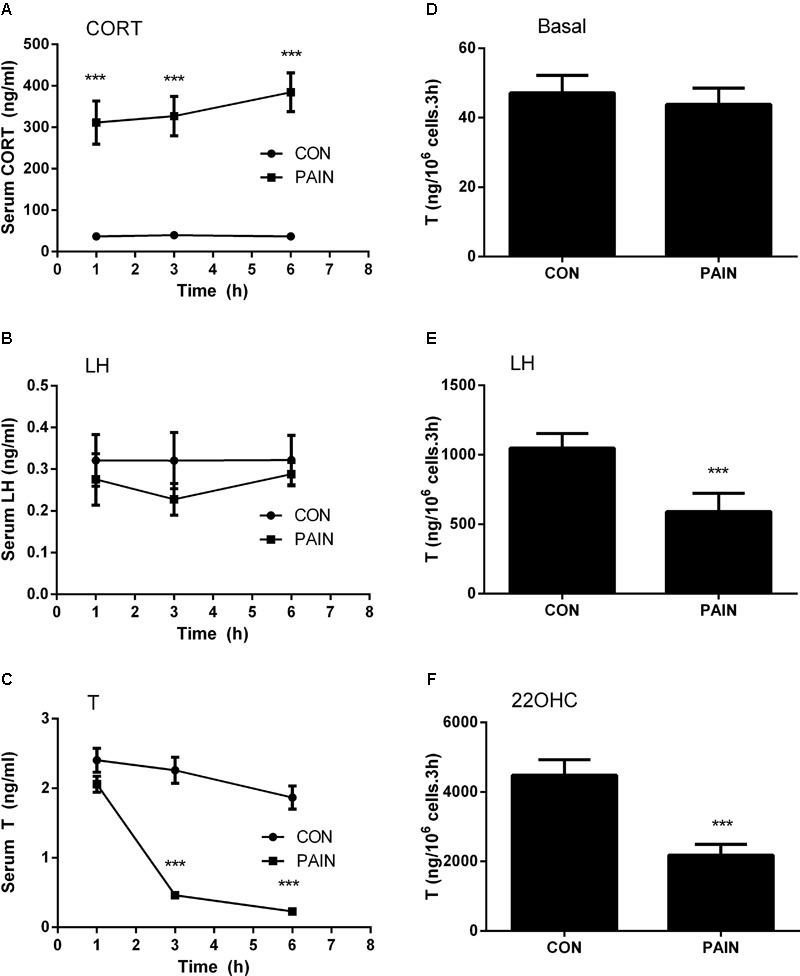
Plasma corticosterone (CORT), luteinizing hormone (LH), testosterone (T) concentrations, and T production of rat Leydig cells during the course of pain. Serum CORT, LH, and T levels were measured at each time point and Leydig cells isolated at hour 3 after pain were stimulated for 3 h without (basal) or with 100 ng/ml LH (LH) or 20 μM 22R-hydroxycholesterol (22OHC). **(A)** Plasma CORT; **(B)** plasma LH; **(C)** plasma T levels; **(D)** basal; **(E)** LH-stimulated; **(F)** 22OHC-mediated T production. Mean values ± SEM, *n* = 6. Asterisks “^∗∗∗^” indicate significant difference when compared to the control (without pain) at each time-point at *P* < 0.001.

The concentrations of plasma CORT in the control group were 36.96 ± 4.70 (mean ± SEM, *n* = 6), 39.66 ± 5.49, and 36.81 ± 6.78 ng/ml at hour 1, 3, and 6 during the course of pain stimulation, respectively, and there were no differences between these groups (**Figure 1A**). This level of plasma CORT conformed to the unstressed level of rats as previously reported (Lin et al., 2014), indicating that the control rats were not under stress condition. During the course of pain stimulation, plasma CORT levels in pain-stimulated groups were significantly elevated at hour 1, 3, and 6, when compared to those from the control rats (**Figure 1A**), indicating that these rats were under stress condition starting at hour 1. Plasma LH levels in pain-stimulated rats did not significantly alter during the course of pain in all groups (**Figure 1B**), indicating that pituitary LH secretion was not affected by pain. Since there was a circadian nature of plasma T, the concentrations of T during the course of pain were compared to the respective control time-point. Plasma T concentrations were significantly reduced when compared to the control group starting at hour 3 (**Figure 1C**), suggesting that the lower T levels followed the increase of plasma CORT levels 2 h later and the primary cause of the reduced T levels was not a result of the change of plasma LH levels.

**FIGURE 2:**
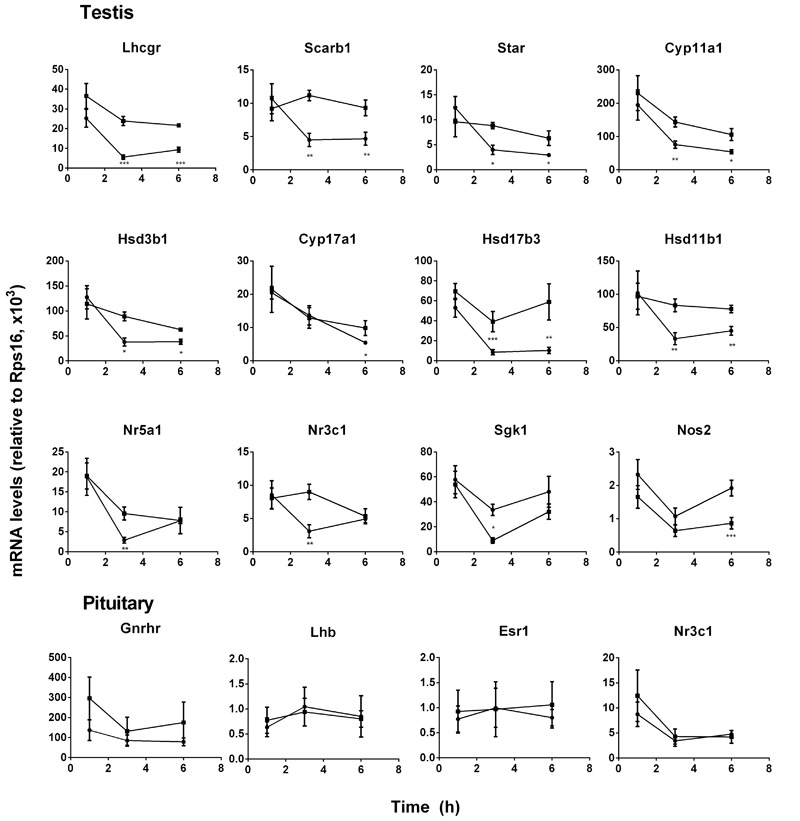
Messenger RNA levels in testis and pituitary during the course of pain. Black circle, control; Black square, pain. Testis genes: *Lhcgr, Scarb1, Star, Cyp11a1, Hsd3b1, Cyp11a1, Cyp17a1, Hsd17b3, Hsd11b1, Nr5a1, Nr3c1, Sgk1*, and *Nos2*; Pituitary genes: *Lhb, Gnrhr, Esr1*, and *Nr3c1*. Mean values ± SEM, *n* = 6. ^∗^, ^∗∗^, and ^∗∗∗^ indicate significant differences when compared to the control (without pain) at each time point at *P* < 0.05, 0.01, 0.001, respectively.

We further purified Leydig cells from the control rats and the pain-stimulated rats at hour 3. Leydig cells were treated without (basal) or with 100 ng/ml LH (for maximal stimulation) (Lin et al., 2014) or 20 μM 22OHC (for replacement of cholesterol as the substrate of CYP11A1) (Lin et al., 2014). Pain did not suppress the basal T production (**Figure 1D**), but significantly suppressed LH-stimulated (**Figure 1E**) and 22OHC-mediated (**Figure 1F**) T production of Leydig cells (**Figure 2**), indicating that the lower plasma levels of T were caused by the defects of Leydig cell steroidogenesis after LH binding as well as the androgen biosynthetic enzymes.

### Gene Expression Levels in Pituitary and Testes

Pituitary LH production depends on the expression level of *Lhb* that was the rate-limiting step for LH (Lin et al., 2014) and its regulatory genes (*Gnrhr, Esr1*, and *Nr3c1*). Pituitary *Lhb* expression was not affected during the course of pain (**Figure 2**, pituitary genes), conforming to plasma LH levels. The regulatory genes, including *Gnrhr, Esr1*, and *Nr3c1*, were not affected by pain (**Figure 2**, pituitary genes). The expression levels of *Lhcgr, Scarb1, Star, Cyp11a1, Hsd3b1, Hsd17b3*, and *Hsd11b1* were significantly lower at hour 3 and 6, while *Cyp17a1* and *Nos2* levels were lower at hour 6 during the course of pain (**Figure 2**, testis genes). Interestingly, *Nr5a1, Nr3c1*, and *Sgk1* were all lower at hour 3 and recovered at hour 6 (**Figure 2**, testis genes). These indicated that the steroidogenesis-related genes in the testis of the pain-stimulated group were downregulated in a different pattern.

### Pain Downregulates STAR, LHCGR, and Steroidogenic Enzymes in Leydig Cells

**FIGURE 3:**
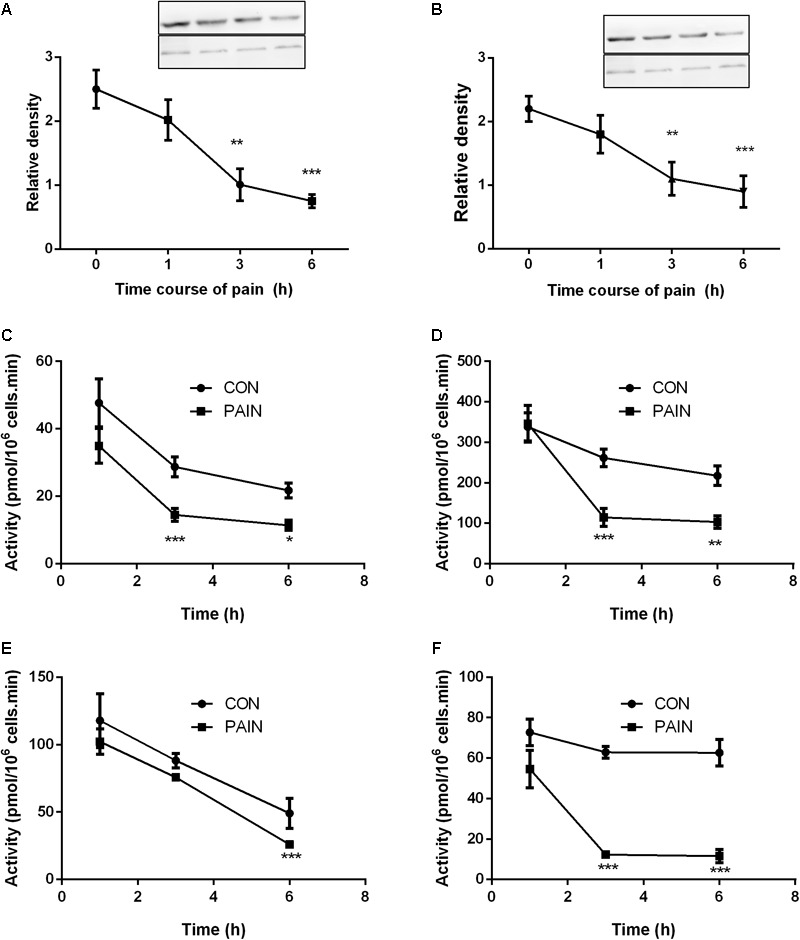
The expression levels of STAR and LHCGR, as well as steroidogenic enzyme activities in the testes from control (CON) and pain-stimulated (PAIN) rats. Protein levels were adjusted by β-actin (ACTB). **(A)** STAR; **(B)** LHCGR; **(C)** CYP11A1; **(D)** HSD3B1; **(E)** CYP17A1; and **(F)** HSD17B3. Mean values ± SEM, *n* = 4–6. Asterisks ^∗^, ^∗∗^, and ^∗∗∗^ indicate significant differences when compared to the control (without pain) at each time point at *P* < 0.05, 0.01, and 0.001, respectively.

The STAR protein was the rate-limiting step for Leydig cell steroidogenesis. LHCGR is the signaling transducer for LH binding and their levels were analyzed by Western blot. As shown in **Figure 3**, both STAR and LHCGR levels were significantly lower at hour 3 and 6 during the course of pain, conforming to the change of their mRNA levels. The steroidogenic enzyme activities for CYP11A1, HSD3B1, CYP17A1, and HSD17B3 were listed in **Figure 3**. CYP11A1, HSD3B1, and HSD17B3 activities were significantly lower at hour 3 after pain stimulation and CYP17A1 was also lower at hour 6 after pain stimulation.

### RU486 Reverses Pain-Induced Decrease of T Levels

**FIGURE 4:**
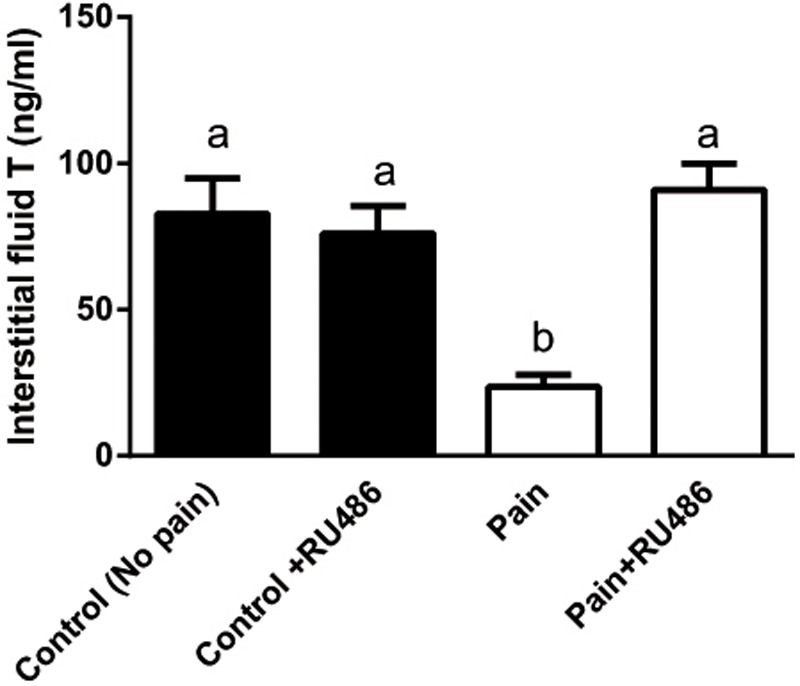
Interstitial fluid T levels of control (CON) and pain (PAIN) rats without or with RU486 (+RU486) treatment at hour 3 during the course of pain stimulation. Rats were assigned to control (No pain), no pain + RU486 (+RU486) treatment, pain (Pain), and pain + RU486 treatment groups. Mean ± SEM, *n* = 6. Identical letters indicate no significant difference between two groups at *P* < 0.05.

Since the elevated CORT levels were followed by the lower T levels in the serum 2 h later during the course of pain, we investigated whether CORT suppressed T production via NR3C1. We injected rats with RU486, an antagonist of NR3C1, and then the rats were subjected to pain stimulation immediately. As shown in **Figure 4**, RU486 significantly reversed the pain-mediated inhibition of T level at hour 3, indicating that pain caused suppression of T production via increase of plasma CORT, which in turn bound to NR3C1 to act.

### DHEA Changes HSD11B1 Catalytic Direction

**FIGURE 5:**
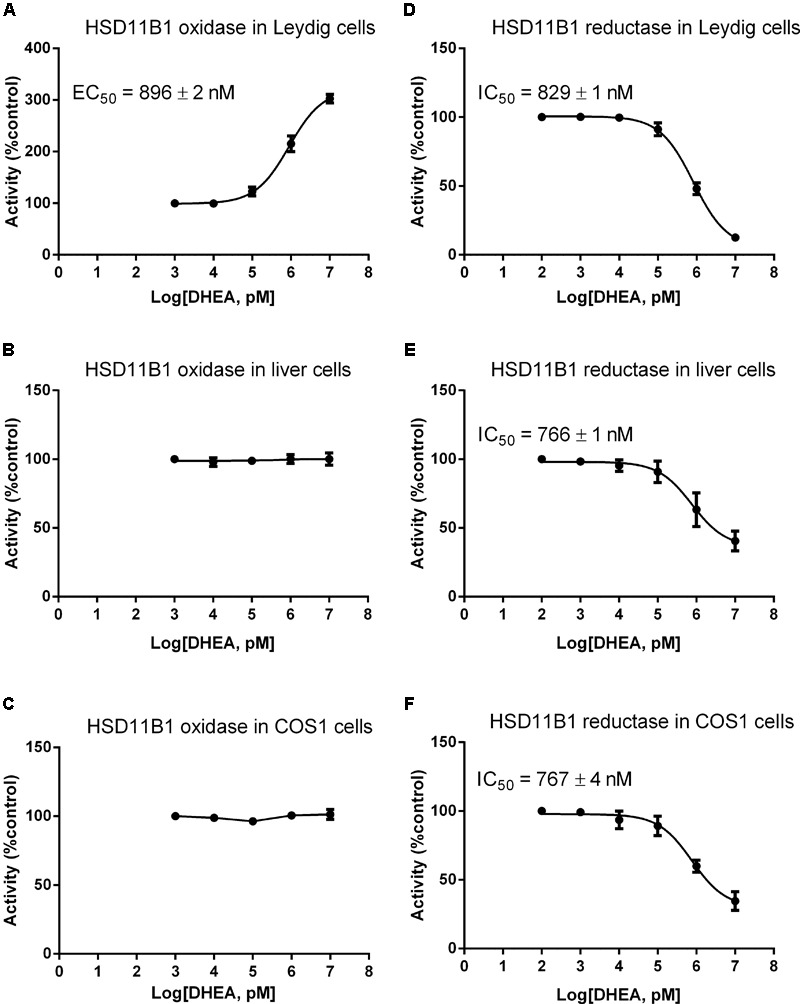
Effects of DHEA on the HSD11B oxidase and reductase activity in Leydig, liver, and COS1-*Hsd11b1* cells. **(A,D)** Leydig cells; **(B,E)** liver cells; **(C,F)** COS1-*Hsd11b1* cells. **(A–C)** HSD11B oxidase; **(D–F)** HSD11B reductase. Mean values ± SEM, *n* = 4.

We asked whether DHEA can directly regulate HSD11B1 direction, because this enzyme is a bidirectional enzyme (Agarwal et al., 1989). We studied the effects of different concentrations of DHEA on HSD11B1 oxidase and reductase in intact rat Leydig, liver, and COS1-*Hsd11b1* cells. As shown in **Figure 5**, DEHA concentration-dependently increased HSD11B1 oxidase activity in intact Leydig cells with EC_50_ value of 896 ± 2 nM (**Figure 5A**) without affecting HSD11B1 oxidase activity in intact liver cells (**Figure 5B**) and intact COS1-*Hsd11b1* cells (**Figure 5C**). DHEA concentration dependently inhibited HSD11B1 reductase activity in intact Leydig (**Figure 5D**), liver (**Figure 5E**), and COS1-*Hsd11b1* cells with IC_50_ values of 829 ± 1, 766 ± 1, and 767 ± 4 nM, respectively. This indicated that the effects of DHEA on HSD11B1 in the liver and Leydig cells were different.

### DHEA Competitively Inhibits HSD11B1 Reductase Activity

**FIGURE 6:**
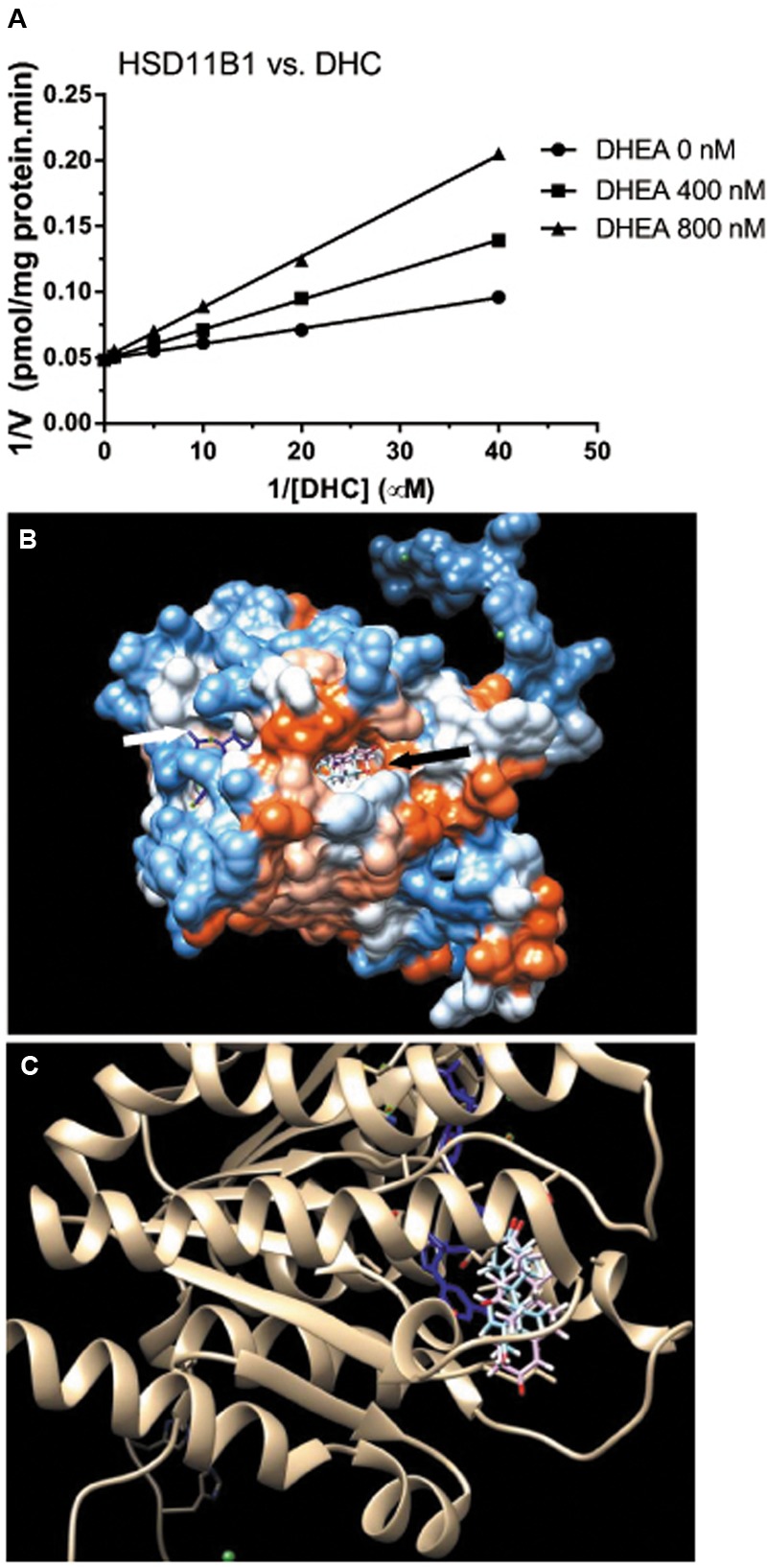
Mode of action of DHEA on HSD11B1 reductase and molecular docking simulation analysis of its interaction with human enzyme. **(A)** Lineweaver–Burk plot of rat 11β-hydroxysteroid dehydrogenase 1 (HSD11B1) reductase versus DHC in the presence of DHEA; **(B)** docking of DHEA on human HSD11B1 which had two openings: one for cofactor NADP^+^/NADPH (white arrow) and the other for steroid substrate or DHEA (black arrow); **(C)** DHEA (blue color) matched HSD11B1 substrate cortisone (pink color) and was interacting with NADP^+^ (blue color).

We then measured the effects of DHEA on HSD11B1 reductase activity in COS1-*Hsd11b1* microsomes. We found that it competitively inhibited HSD11B1 reductase activity when the substrate DHC was added (**Figure 6A**). We further used the docking software to analyze the binding site of DHEA on human HSD11B1, and the crystal structure of the enzyme was obtained from the Protein Data Bank (PDB id 2ILT; Sorensen et al., 2007). We found that human HSD11B1 had two openings for chemicals, one being the steroid-binding site and the other being the NADP^+^/NADPH-binding site (**Figure 6B**), and that DHEA bound to the steroid-binding site of human HSD11B1. According to the literature (Hosfield et al., 2005), the steroid-binding pocket contained T124, L126, A172, Y177, V180, Y183, L215, L217, T222, and V227 with Y183 as the catalytic residue. The cortisone was found to bind to the steroid-binding pocket with 11keto facing to Y183 residue of the HSD11B1 and have a free energy of -9.13 kcal. Molecular docking simulation analysis for DHEA demonstrated that it bound to the steroid-binding pocket and it contacted Y183 residue. DHEA had a free energy of -8.54 kcal, indicating that it was a potent inhibitor of HSD11B1.

### DHEA Reverses the Pain-Induced T Reduction

**FIGURE 7:**
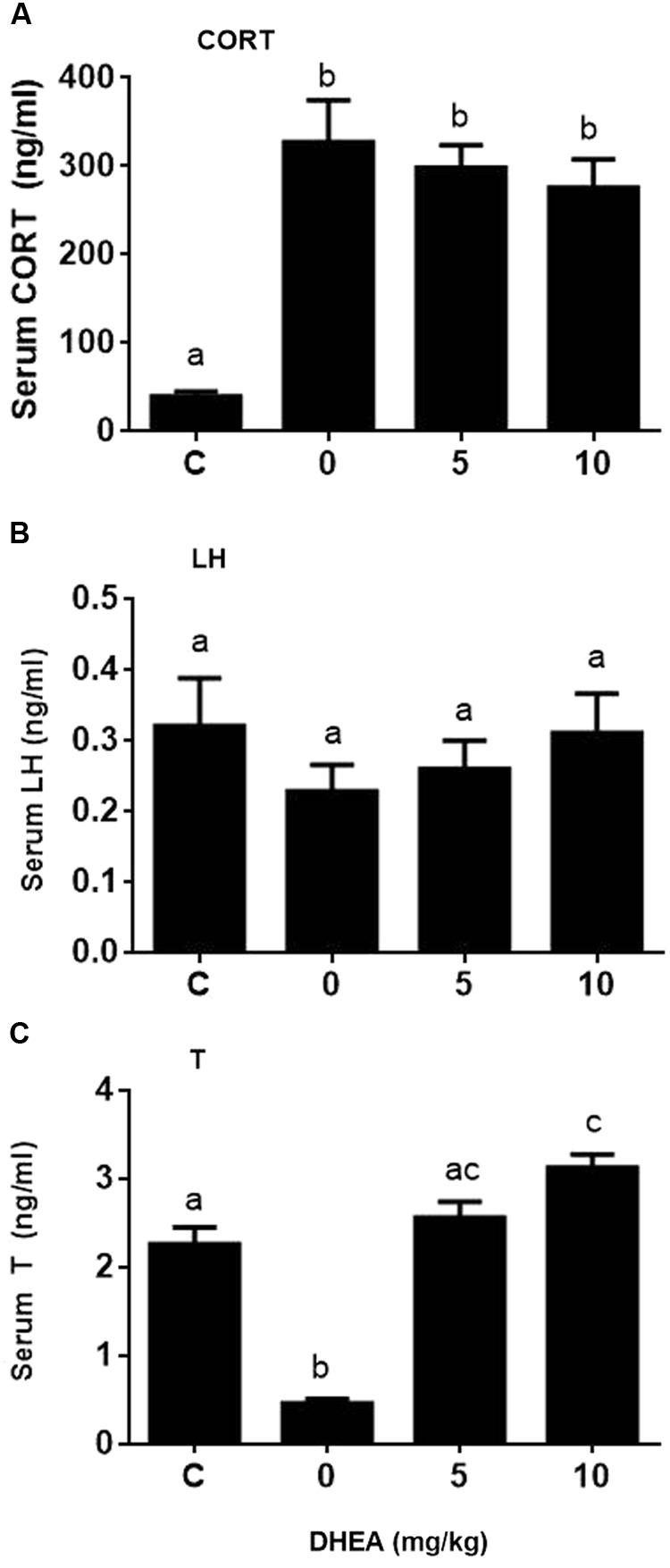
Plasma corticosterone (CORT), luteinizing hormone (LH), and testosterone (T) concentrations at hour 3 of pain stress and the effects of DHEA. Plasma CORT, LH, and T levels were measured at hour 3 after pain were stimulated. **(A)** Plasma CORT; **(B)** plasma LH; **(C)** plasma T levels. Mean values ± SEM, *n* = 6. Identical letters indicate no significant difference between two groups at *P* < 0.05.

**FIGURE 8:**
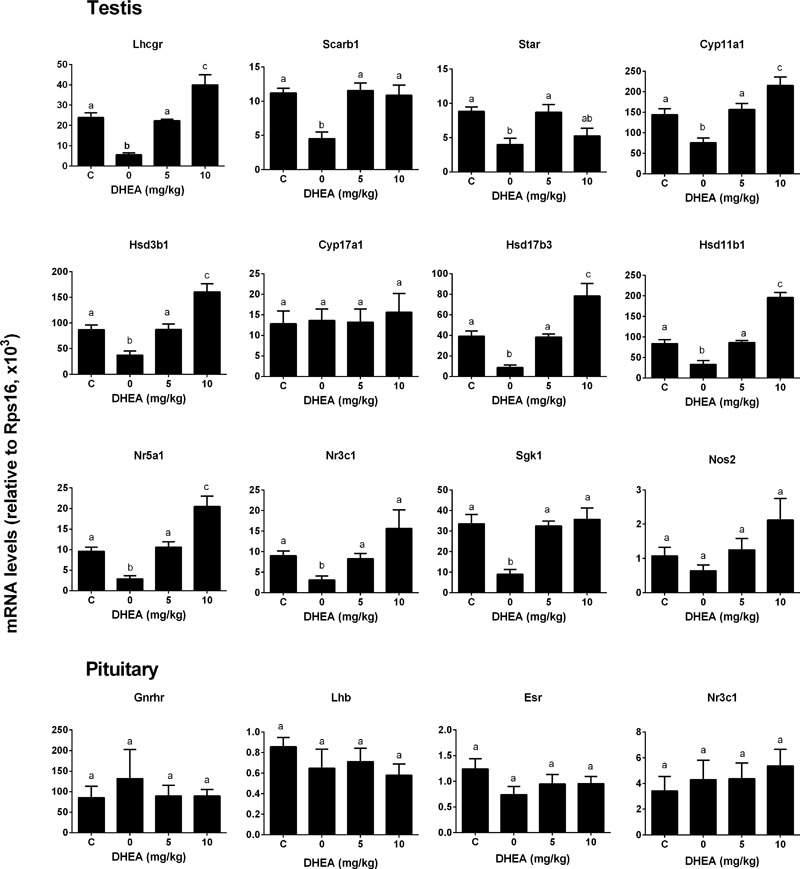
Messenger RNA levels in testis and pituitary in the pain-stressed rats after DHEA treatment. Testis genes: *Lhcgr, Scarb1, Star, Cyp11a1, Hsd3b1, Cyp11a1, Cyp17a1, Hsd17b3, Hsd11b1, Nr5a1, Nr3c1, Sgk1*, and *Nos2*; Pituitary genes: *Lhb, Gnrhr, Esr1*, and *Nr3c1*. Mean values ± SEM, *n* = 6. Identical letters indicate no significant difference between two groups at *P* < 0.05.

We checked whether DHEA could prevent the pain-induced T reduction. We treated rats with DHEA (5 or 10 mg/kg) and 0.5 h later, these rats were subjected to pain stimulation. Indeed, compared to the normal control, pain stimulation increased plasma CORT levels significantly (**Figure 7A**). However, the treatment of DHEA did not affect the total plasma CORT levels (**Figure 7A**). DHEA treatment did not alter plasma LH levels either (**Figure 7B**). However, pain stimulation reduced plasma T levels and 5 and 10 mg/kg DHEA treatment significantly reversed pain-induced reduction of plasma T levels (from 0.46 ± 0.051 to 2.56 ± 0.18 and 3.13 ± 0.15 ng/ml, respectively) (**Figure 7C**). This indicated that DHEA can act locally to reverse pain-induced reduction of testosterone production. We further examined the testis and pituitary gene expression without or with DHEA for the acute stress model. Again, DHEA did not alter pituitary *Lhb, Gnrhr, Esr1*, and *Nr3c1* levels (**Figure 8**, pituitary genes), suggesting that the acute treatment of DHEA did not alter pituitary function. DHEA reversed the expression levels of *Lhcgr, Scarb1, Star, Cyp11a1, Hsd3b1, Hsd17b3*, and *Hsd11b1* in the testis at hour 3 after pain (**Figure 8**, testis genes).

## Discussion

In the present study, we demonstrated that acute pain stimulation to male adult rats caused the elevated plasma CORT level as early as 1 h under pain stimulation. The plasma T levels were significantly lowered after 3 h of being subjected to pain stimulation. The decrease of T levels followed downregulation of LH signaling (*Lhcgr*), cholesterol transporter (*Scarb1* and *Star*), and steroidogenic enzyme (*Cyp11a1, Hsd3b1*, and *Hsd17b3*) expression. DHEA was found to regulate HSD11B1 direction by inhibiting HSD11B1 reductase activity, thus lowering the intracellular CORT levels in Leydig cells to reverse CORT mediated reduction of T.

The elevation of plasma CORT level after acute pain stimulation indicated that rats had stress responses. This conformed to the significant increase in total plasma CORT levels in other types of stresses in rats, such as immobilization and psychological stresses (Blanchard et al., 1995; Spencer et al., 1996; Lin et al., 2014). In rats, the level of the bioactive GC relies on the circulating CORT level, the plasma CORT binding globulin level, and local CORT metabolism by HSD11B1 in rat Leydig cells. In rat Leydig cells, HSD11B1 is the primary isoform of HSD11B family (Ge et al., 2005a), acting as a bidirectional oxidoreductase in which the oxidase activity can be dictated by the prevailing redox potential of NADP/NADPH levels (Ge et al., 1997a; Ge and Hardy, 2000), which protect Leydig cells from the elevation of plasma CORT level (Monder et al., 1994b). The other isoform, an NAD-dependent HSD11B2, which acts as a unidirectional oxidase, accounted for the 0.1% of total amount of Leydig cell HSD11B activity (Ge et al., 2005a). In the present study, we observed that *Hsd11b1* expression level was significantly downregulated at hour 3 and later during the course of pain, indicating that the protection of HSD11B1 was inhibited. Thus, CORT excess with the lower *Hsd11b1* could increase intracellular level of CORT, which inhibited Leydig cell steroidogenesis. Interestingly, there was a decreased expression from *Nr3c1* at hour 3, indicating that there was an initial protection of excess of CORT. However, by 6 h, *Nr3c1* returned to the control level. It has been demonstrated that GCs inhibited Leydig cell steroidogenesis by acting on the promoters of the genes of many steroidogenesis-related proteins, including *Cyp11a1* (Hales and Payne, 1989), *Star* (Martin and Tremblay, 2008; Xiao et al., 2010), and *Hsd3b1* (Xiao et al., 2010). Since STAR is the rate-limiting protein for steroidogenesis in Leydig cells (Stocco and Clark, 1996; Stocco, 2001), the downregulation of its expression led to the suppression of androgen biosynthesis. Martin and Tremblay (2008) found that GC inhibited *Star* expression partially via the suppression of NR4A1-dependent transactivation of the *Star* promoter in mouse MA-10 Leydig cells. Interestingly, the 3-h pain stimulation also caused the downregulation of *Nr5a1*, the critical transcription factor for the expression of many steroidogenesis-related genes. However, *Nr5a1* returned to the normal level after 6 h of pain.

The plasma T levels of the stressed rats were decreased significantly by 3-6 h during the course of pain (**Figure 1C**). The decrease of plasma T levels may be due to the reduction of production of T in Leydig cells, as plasma LH levels were not altered. Therefore, CORT appears to directly suppress Leydig cell steroidogenesis. We observed the downregulation of cholesterol transportation system (*Scarb1* and *Star*) and steroidogenic enzyme (*Cyp11a1, Hsd3b1*, and *Hsd17b3*) at hour 3 and later. The downregulation of many steroidogenesis-related genes including the *Star* at hour 3 after pain stimulation might explain the reduction of T biosynthesis. After 3-6 h during the course of pain, *Lhcgr* level was significantly downregulated, conforming to the lower T production of Leydig cells after LH stimulation (**Figure 2**).

In the present study, pain stimulation caused significant elevation of CORT, indicating a stress condition during the course of pain. Several other factors that induce stress may also affect the suppression of T biosynthesis. These factors included testicular opioids (Akibami and Mann, 1996) and NOS (Kostic et al., 1998). However, the role and importance of testicular opioids and NOS in the stress-mediated suppression of T production is still under debate. Knockout of several NOS isoforms did not antagonize the acute stress-mediated action on T production under the immobilization stress (Weissman et al., 2007, 2009), suggesting that NOS signals in the acute stress-mediated action was minimal. Indeed, in the present study, we also failed to find any significant increase of expression level of the inducible NOS (*Nos2*) (**Figure 2**). Instead, *Nos2* expression was downregulated at hour 6 (**Figure 2**). Acute stress-induced suppression of T production in the Leydig cells could be mediated by NR3C1. Indeed, the intratesticular injection of NR3C1 antagonist RU486 completely prevented the interstitial fluid T level suppression in the stressed rats (**Figure 4**), suggesting that CORT acted via the NR3C1 in Leydig cells. This antagonist was also observed in rats and mice under immobilization stress (Dong et al., 2004; Lin et al., 2014). We also measured the expression levels of *Nr3c1* and its target gene *Sgk1*, and found that there was a decreased expression level of *Nr3c1* at hour 3 under the stress (**Figure 4**), suggesting a compensatory mechanism to minimize CORT action at hour 3 (Monder et al., 1994b). However, the persistent pain stimulation caused their values to return to the unstressed levels (**Figure 2**).

DHEA and its sulfate ester DHEA-S are the most abundant steroids in the human circulation (Feher and Bodrogi, 1982). Previous studies have demonstrated that the expression of HSD11B1 in murine adipocytes (Apostolova et al., 2005) and rat liver (Gu et al., 2003) was downregulated by DHEA, thus leading to the lower oxidoreductase activity of HSD11B1. However, in the present study, we found that DHEA actually reversed pain-mediated reduction of *Hsd11b1* (**Figure 8**). This difference could be due to the cell-specific effects of DHEA. However, in the present study, we demonstrated that DHEA potently inhibited HSD11B1 reductase activity in Leydig, liver, and COS1-*Hsd11b1* cells (**Figure 5**) without affecting HSD11B1 oxidase activity in liver and COS1-*Hsd11b1* cells, thus inhibiting the reactivation of inert GCs into active GCs. This inhibition that DHEA exerted was competitive against the steroid substrate (**Figure 6**). The docking study suggested that DHEA had a higher binding affinity with the steroid-binding site of human HSD11B1 (a free energy of -8.54 kcal) (**Figure 6**). Interestingly, DHEA significantly increased HSD11B oxidase activity in rat Leydig cells (**Figure 5**). This action was not the same for HSD11B oxidase in liver and COS1-*Hsd11b1* cells. One explanation is the presence of minor HSD11B2 in rat Leydig cells, although its expression level in Leydig cells was only 0.1% that of *Hsd11b1* (Ge et al., 2005a). Indeed, using an antisense probe to downregulate *Hsd11b1*, the HSD11B oxidase was significantly increased (Ge et al., 2005a), indicating that this HSD11B2 oxidase activity was dominant after the downregulation of *Hsd11b1*. Increased HSD11B oxidase in Leydig cells clearly inactivated local GC levels even though the circulating CORT was still higher and was not altered after DHEA treatment. Another possibility is that the conversion of DHEA to androstenedione alters the redox potential favoring oxidase activity. HSD11B1 is a bidirectional oxidoreductase NADP^+^/NADPH dependent enzyme (Sandeep and Walker, 2001). Whether it will operate as oxidase or as reductase is mainly dictated by the particular cellular environment of redox potential, defined by NADP^+^/NADPH ratio. This redox potential is dictated by the concentration of substrates and the presence of enzymes utilizing and competing for NADP^+^ or NADPH cofactors. In Leydig cells, at equilibrium, more NADP^+^ is produced than NADPH, because there are more enzymatic reactions (such as CYP11A1, CYP17A1, and HSD17B3) that utilize NADPH to yield more NADP^+^. Thus, under these conditions, HSD11B1 operates in the oxidative mode, inactivating GCs generating NADPH that couples with androgen biosynthetic reactions and promoting testosterone synthesis. Provided there is enough substrate for HSD11B1 like CORT, that will operate in a cyclic manner coupled with androgen biosynthetic pathway.

In the presence of DHEA, in Leydig cells, DHEA is rapidly converted to androstenedione by the action of NAD^+^-dependent HSD3B. Androstenedione being a substrate of HSD17B3, it is converted to testosterone. There are at least three enzymes involved in the metabolism of T utilizing three NADPH and generating three NADP^+^. Abundance of NADP^+^ cofactor stimulates HSD11B1 in oxidase direction. So, DHEA accelerated oxidase activity in Leydig cells, but not in the liver or transfected COS cells because there is no such enzymatic machinery to utilize NADPH. In liver or COS cells, NADPH is generated by glucose-6-phosphate dehydrogenase and with the redox potential being reversed (Ge and Hardy, 2000; Atanasov et al., 2004), HSD11B1 mostly operates in a reductase direction, inactivating DHC to CORT. In addition to accelerating HSD11B1 in the oxidase direction, DHEA is clearly shown as a competitive inhibitor of HSD11B1 reductase in the microsomal fraction. So, in the presence of DHEA, two factors are working against reductase in all three cell types: the inhibitor of reductase and in Leydig cells, lack of NADPH availability to carry on the reductase reaction.

In summary, acute pain stimulation led to the elevated CORT levels as early as 1 h which is followed by the reduced T levels 3 h after acute pain stimulation. High CORT level may bind to NR3C1, which blocked LH signaling (*Lhcgr*), cholesterol transport genes (*Scarb1* and *Star*), and steroidogenic enzyme (*Cyp11a1, Hsd3b1*, and *Hsd17b3*) gene expression. Inhibition of the reductase of HSD11B1 by DHEA makes the minor HSD11B2 as the dominant isoform which is capable of eliminating local GCs by its oxidase activity.

## Author Contributions

QZ, XL, FG, MY, and R-SG designed the study. QZ, XL, FG, H-SD, MX, TB, JL, YW, and YS performed the experiments. R-SG analyzed the data. XL and R-SG wrote the manuscript.

## Conflict of Interest Statement

The authors declare that the research was conducted in the absence of any commercial or financial relationships that could be construed as a potential conflict of interest.

## Footnotes

1 http://pubchem.ncbi.nlm.nih.gov
