# Supplementary material for: Dehydroepiandrosterone Antagonizes Pain Stress-Induced Suppression of Testosterone Production in Male Rats
Source: Front Pharmacol. 2018 Apr 16;9:322. doi: 10.3389/fphar.2018.00322 (PMC5911460; doi:10.3389/fphar.2018.00322)
Supplement: Supplementary file 1 [file Table_1.DOCX]

**Supplemental Table 1 The information for gene names, primers, and their Ct values**

| **Gene** | **Accession no.** | **Forward primer** | **Reverse primer** | **Ct** |
| --- | --- | --- | --- | --- |
| **Testis** | | | | |
| Rps16 | X17665 | 5’AAGTCTTCGGACGCAAGAAA3’ | 5’TGCCCAGAAGCAGAACAG3’ | 20.7 |
| Lhcgr | NM_012978 | 5’CTGCGCTGTCCTGGCC3’ | 5’CGACCTCATTAAGTCCCCTGAA3’ | 26.7 |
| Scarb1 | NM_031541 | 5’ATGGTACTGCCGGGCAGAT3’ | 5’CGAACACCCTTGATTCCTGGTA3’ | 26.5 |
| Star | NM_031558 | 5’CCCAAATGTCAAGGAAATCA3’’ | 5’AGGCATCTCCCCAAAGTG3’ | 26.3 |
| Hsd11b1 | NM_017080.2 | 5’TCTTCTTGGCCTACTACTAC3’ | 5’TTGCTGGCCCCTGTGACAAT3’ | 24.5 |
| Sgk1 | NM_019232 | 5’CAGAATGAGGGGAATGGTAGC3’ | 5’TTGGCGTGGGGATTTGAG3’ | 26.8 |
| Nos2 | NM_012611.3 | 5’AATAGAGGAACATCTGGCCAGG | 5’ACTTCCTCCAGGATGTTGTA3’ | 27.5 |
| Cyp11a1 | NM_017286 | 5’AAGTATCCGTGATGTGGG3’ | 5’TCATACAGTGTCGCCTTTTCT3’ | 27.1 |
| Hsd3b1 | NM_017265 | 5’CCCTGCTCTACTGGCTTGC3’ | 5’CCCTGCTCTACTGGCTTGC3’ | 25.7 |
| Cyp17a1 | NM_012753 | 5’TGGCTTTCCTGGTGCACAATC3’ | 5’TGAAAGTTGGTGTTCGGCTGAA3’ | 26.6 |
| Hsd17b3 | NM_054007 | 5’ TTTCTTCGGGAGTAGGGGTTC3’ | 5 TCATCGGCGGTCTTGGTCG3’ | 28.8 |
| Nr5a1 | NM_053344 | 5’CAGAGCTGCAAAATCGACAA3’ | 5’ CCCGAATCTGTGCTTTCTTC 3’ | 26.2 |
| Nr3c1 | NM_012576 | 5’GAAATGGGCAAAGGCGATAC3’ | 5’GCAAATGCCATGAGAAACAT3 | 27.0 |
| **Pituitary** | | | | |
| Rps16 | X17665 | 5’AAGTCTTCGGACGCAAGAAA3’ | 5’TGCCCAGAAGCAGAACAG3’ | 20.5 |
| Lhb | NM_008497 | 5’CTGCTGCTGAGCCCA AGTGT-3’ | 5’TGCTGGTGGTGAAGGTGATG3’ | 30.7 |
| Gnrhr | U92470 | 5’CTTGAAGCCCGTCCTTGG-3’ | 5’GCGATCCAGGCTAATCAC3’ | 23.5 |
| Esr1 | NM012689 | 5’GCTCCAATTCTGACAATCG3’ | 5’TTTCGTATCCCGCCTTTCA3’ | 25.6 |
| Nr3c1 | NM_012576 | 5’GAAATGGGCAAAGGCGATAC3’ | 5’GCAAATGCCATGAGAAACAT3’ | 25.9 |
